# Loss of BRCA1 Spontaneously Induces the Tumorigenesis in Lacrimal Gland

**DOI:** 10.1155/2018/8120579

**Published:** 2018-12-17

**Authors:** Sun Eui Kim, Hye Jung Baek, Eun Jung Park, Sung Chul Lim, Sang Soo Kim

**Affiliations:** ^1^Research Institute, National Cancer Center Research Institute, Goyang 10408, Republic of Korea; ^2^Department of Pathology, College of Medicine, Chosun University, Gwangju 61452, Republic of Korea

## Abstract

Environmental and genetic factors exert important influences on lifespan and neoplastic transformation. We have previously shown that spontaneous tumors form frequently in mice homozygous for a full-length *Brca1* deletion. In general, mutations of BRCA1 are closely associated with induction of breast and ovarian cancers but are also known to contribute to the incidence of other cancers at a low frequency. Female *Brca1*-mutant mice (*Brca1^co/co^MMTV-cre*) were generated by crossing *Brca1* conditional knockout mice and *MMTV-cre* mice, and the occurrence of lacrimal gland abnormalities and tumors was followed until mice reached 18 months of age. Lacrimal gland tumors, which occur at a very low frequency in the human population (1 per 1,000,000 per year), were detected in 7 cases of *Brca1^co/co^MMTV-cre* mice (2.75%) older than 9 months of age. None of seven mice exhibited any abnormality in the mammary gland including neoplasia, suggesting lacrimal gland tumor is spontaneously and independently formed. These tumors, which were detected in seven mutant mice that displayed exophthalmoses, were malignant, originated from epithelial cells, and were identified as acinic cell carcinoma by pathological analysis. Further analysis revealed that tumorigenesis was accompanied by the accumulation of cyclin D1 and decreased expression of the cellular oncogenes, c-Myc, c-Jun, and c-Raf. Tumors also exhibited rearrangement of cytoskeletal proteins, including *β*-catenin, keratin 5, and vimentin, depending on tumor progression. These results suggest that BRCA1 is involved in genetic stability of the lacrimal gland, providing new insight into genomic instability in organism maintenance and tumorigenesis of the lacrimal gland.

## 1. Introduction

The loss of BRCA1, one of the most widely studied tumor-suppressor proteins, is transmitted hereditarily. BRCA1 functions have been extensively investigated in multiple organisms, particularly in mice, where gene targeting has been applied to introduce a number of different mutations into *Brca1* (reviewed [1]). Depending on the mutation, *Brca1*-mutant mice exhibit various abnormal phenotypes, including developmental defects, growth retardation, apoptosis, premature senescence, DNA damage repair abnormalities, centrosome amplification, deregulation of the cell cycle, and genetic instability [2, 3]. In the *BRCA1*-mutant carrier, breast and ovarian cancers are the predominant abnormalities in whole-body haploinsufficiency. However, several reports have described a significantly increased risk of pancreatic, prostate, colorectal, esophageal, liver, stomach, and uterine cancers with alterations in BRCA1 [4, 5].

Lacrimal gland tumors are very rare in humans, with an estimated incidence of 1 per 1 million individuals per year [6]. Although many types of tumors have been identified in the lacrimal gland, they can be roughly divided into those that originate in epithelial cells and those with a nonepithelial origin. Notably, about half of epithelial-based lacrimal gland lesions are malignant [7]. The primary treatment for benign lacrimal gland tumors is surgical resection, whereas malignant tumors are generally removed surgically followed by radiotherapy and/or chemotherapy [8]. Hadron beam therapy has also been considered a nonsurgical treatment option for patients not suitable for surgical treatment [9]. However, there is some controversy surrounding therapeutic options, particularly for malignant tumors. Although the prognosis for patients with benign type tumors is generally good, malignant lacrimal gland tumors are often aggressive and tend to recur frequently, resulting in a lower survival rate [8]. However, because of the limited number of cases and few opportunities to clinical attempt, few large improvements have been made in its prevention or treatment.

Here, based on an investigation of seven cases of lacrimal tumors from *Brca1*-mutant mice, we report the presence of acinic cell carcinomas in the lacrimal gland and establish the characteristics of BRCA1-associated lacrimal tumors by comparing them with those of BRCA1-associated breast cancer and typical lacrimal tumors.

## 2. Materials and Methods

### 2.1. Animal Experiments

*Brca1* conditional knockout and *MMTV-* (mouse mammary tumor virus-) *cre* transgenic mice were provided by the National Cancer Institute (NCI, USA) mouse repository. Female *Brca1*-mutant mice (*Brca1^co/co^MMTV-cre*) were generated by crossing *Brca1* conditional knockout mice, with *MMTV-cre* mice, originally generated by Dr. Deng and Dr. Hennighausen, respectively [10, 11]. Mutant mice and wild-type littermates were examined weekly for evidence of abnormalities or tumors up to 18 months of age. In mice that developed symptoms, tissues were collected, divided, frozen in liquid nitrogen, and stored at −80°C or fixed in 10% (*v*/*v*) buffered formalin. Tumor status of a 10-month-old mouse was examined by acquiring tumor images using a 7-T MR imager (Bruker BioSpec, Rheinstetten, Germany) from anesthetized mice by inhalation of isoflurane (2.0% isoflurane in air). Tumor volume was calculated according to the formula, *V* = (*L* × *W* × *D* × *π*)/6, where *V* is volume, *L* is length, *W* is width, and *D* is depth. All procedures involving animals and their care were approved by the Institutional Animal Care and Use Committee of the National Cancer Center (Goyang, Korea) followed by the Guide for the Care and Use of Laboratory Animals (US National Research Council).

### 2.2. Histology and Immunohistochemical Staining

For histology, dissected tissues were immediately fixed in 10% (*v*/*v*) formalin and embedded in paraffin. Tumor sections (5 *μ*m) were deparaffinized and rehydrated in xylene and graded ethanol series. Antigen retrieval was performed at 125°C for 3 min by a decloaking chamber (Biocare Medical, Pacheco, CA, USA) in target retrieval solution (Dako, Santa Clara, CA, USA). Antigenic proteins were detected using a Zymed Histostain kit (Invitrogen-Thermo Fisher, Waltham, MA, USA) according to the manufacturer's instructions. The following primary antibodies (1 : 100 dilution) were used: anti-cyclin D1 (#2978), anti-vimentin (#5741), anti-*β*-catenin (#8480), (all from Cell Signaling Technology, Danvers, MA, USA), anti-keratin 5 (#905504, BioLegend, San Diego, CA, USA), and anti-PCNA (#HPA030522, Atlas Antibodies, Bromma, Sweden). Hematoxylin and eosin (H&E) stainings were performed with automated H&E stainer (ST5010 Autostainer XL, Leica Biosystems, Wetzlar, Germany).

### 2.3. Immunoblotting

Western blot analyses were carried out according to standard procedures using enhanced chemiluminescence detection (Pierce-Thermo Fisher, Waltham, MA, USA). In brief, tumor tissues were washed three times with ice-cold phosphate-buffered saline (PBS) and were extracted with handheld homogenizer in the buffer consisting of 50 mM Tris-HCl (pH 7.5), 10% glycerol, 1% Triton X-100, and 1x protease inhibitor cocktail (Roche, Penzberg, Germany). After centrifugation at 15000 x *g* for 30 min, the supernatants were collected and protein concentrations were determined by the Bradford method with BSA as a standard. The samples were then electrophoresed on 4–20% (*w*/*v*) Novex Tris-Glycine minigels (Invitrogen-Thermo Fisher, Waltham, MA, USA). The separated proteins were transferred to PVDF membranes (Merck-Millipore, Darmstadt, Germany), then blocked with PBS/0.1% Tween 20 (PBST) containing 3% bovine serum albumin (Fraction V). Primary antibody bindings (1 : 1000 dilution) were performed overnight at 4°C. Horseradish peroxidase-conjugated goat anti-rabbit antibody (Jackson Immuno Research, West Grove, PA, USA) was used as a secondary antibody. Membranes were immersed with SuperSignal West Pico Chemiluminescent Substrate (Pierce-Thermo Fisher, Waltham, MA, USA), exposed to Hyperfilm ECL (Amersham-GE Heathcare, Chicago, IL, USA), and then developed. The following primary antibodies were used: anti-c-Myc (#9402), anti-c-Jun (#2361), anti-c-Raf (#9421), anti-JNK (#4668), anti-cyclin D1 (#2978), anti-vimentin (#5741), anti-*β*-actin (#8457) (all from Cell Signaling Technology, Danvers, MA, USA), and anti-PCNA (#HPA030522, Atlas Antibodies, Bromma, Sweden). The primary antibodies applied in this study were reacted to mouse proteins and generated in rabbit to avoid the cross-reactivity with the mouse blood in the tumor tissues.

## 3. Results

### 3.1. Identification of Lacrimal Gland Tumors in Brca1-Mutant Mice

**Figure 1:**
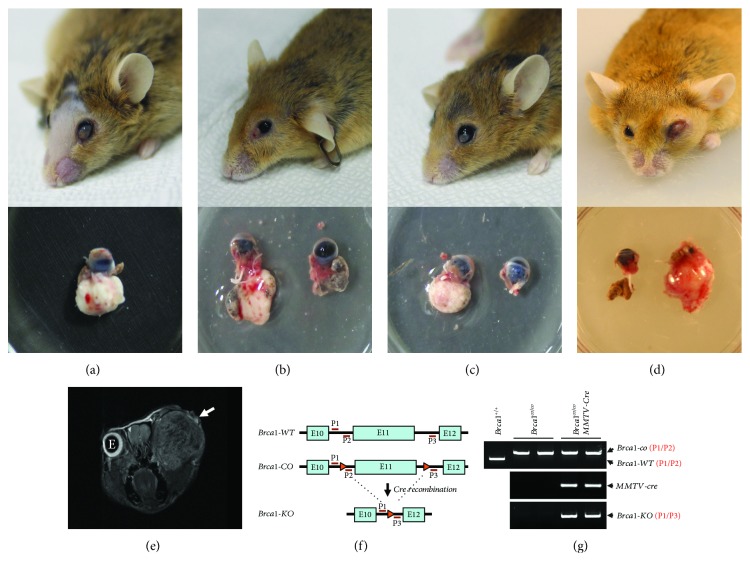
Abnormal eye phenotypes in *Brca1^co/co^MMTV-cre* mice. (a–d) *Brca1^co/co^MMTV-cre* mice that developed tumors at 9 (a), 16 (b), 17 (c), and 10 (d) months of age. Top: the mice exhibited exophthalmoses, protrusions around the eye, and opaque eyeballs. Bottom: dissected eyes and eye-associated tumors from the corresponding *Brca1^co/co^MMTV-cre* mice. (e) Representative MR scan image of *Brca1^co/co^MMTV-cre* mouse that developed a tumor at 10 months of age. Lacrimal tumor (indicated by arrow) side was expanded to the eye region while eye (e) in other side was intact. (f) Targeting construction of *Brca1* deletion by cre recombinase. P1, P2, and P3 represent the primers for detection of targeted mutation and deletion. (g) PCR analysis against *Brca1* using primers as indicated by the numbers. PCR products specific for *Brca1* knockout (P1/P3) were detected in lacrimal tumors but not in normal lacrimal gland.

For the studies of BRCA1-associated breast cancer, we generated the female *Brca1* conditional knockout mice with *MMTV-cre*-mediated recombination (*Brca1^co/co^MMTV-cre*). The cohort of *Brca1*-mutant mice consists of 254 individuals. Mice were maintained in a specific pathogen-free facility with a HEPA-filter-containing air ventilation cage system. Of these female mice, currently seven (2.75%) exhibited exophthalmoses, swelling around the eye, and an opaque eyeball in a single side of the eye, whereas none of their wild-type littermates (*Brca1^co/co^* or *Brca1^+/co^MMTV-cre*) exhibited a similar phenotype over the same period. At the same period, *Brca1^co/co^MMTV-cre* mutant mice showed a high incidence of mammary tumors (56.7%). However, these seven mice with lacrimal tumor did not exhibit any other abnormality including mammary tumor. These phenotypes manifested when mice were 9, 10, 11, 12, 14, 16, and 17 months old, ages that correspond to the initiation of menopause or the postmenopause period. The 9, 10, and 16 months of age mice developed a tumor mass behind the eyeball in each case (Figures 1(a)–1(d)). To further assess these eye abnormalities, we scanned the 10-month-old mouse with exophthalmos by magnetic resonance (MR) imaging. A tumor with a size of 7.4 × 8.6 × 9.0 mm was identified in the eye region; the shape and location of the eye-associated tumor were confirmed upon dissection (Figure 1(e)). To confirm the loss of *Brca1* in lacrimal tumor, we examined the deletion of *Brca1* in the DNA of lacrimal tumors with normal lacrimal glands from wild-type mice as control (Figure 1(f)). The analysis of genomic DNA showed that loss of Brca1 was exclusively detected in the tumor tissues from mutant mice but not in the normal lacrimal gland of wild-type mice (Figure 1(g)).

**Figure 2:**
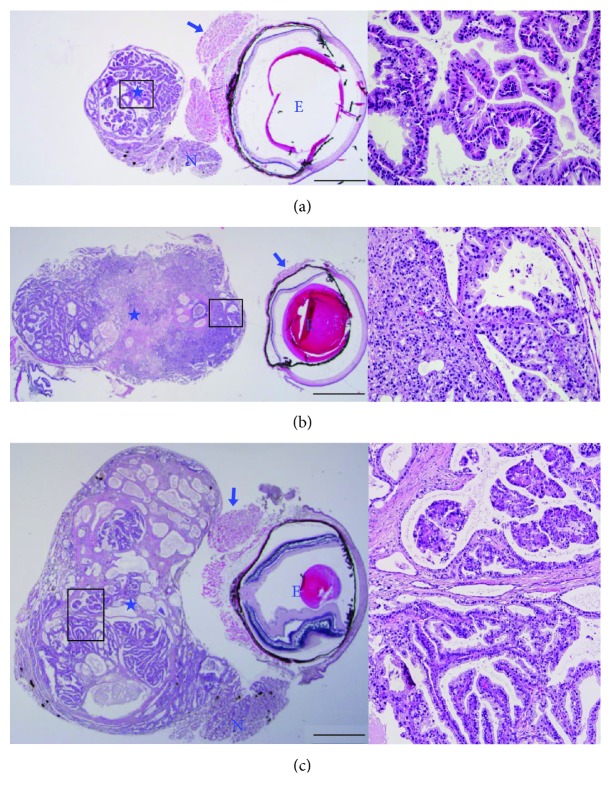
Identification of lacrimal gland tumors in *Brca1^co/co^MMTV-cre* mice. (a–c) Low-power view of H&E-stained sections of the 16 (a), 17 (b), and 9 (c) months of age cases of retrobulbar tumors (asterisks) separated by skeletal muscles (arrows) in the eyeballs (E). Nonneoplastic lacrimal glands (N) adjacent to the tumors are evident. The panels at the right are magnifications of the boxed areas in the adjacent panels. Scale bars: 1 mm.

**Figure 3:**
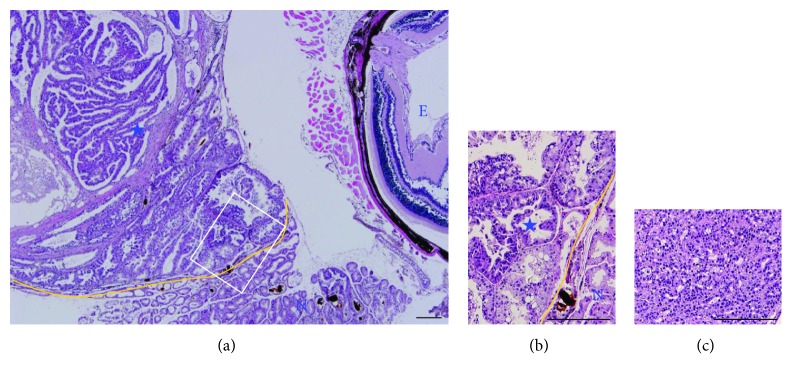
Histological analysis of lacrimal tumors in *Brca1^co/co^MMTV-cre* mice. (a) High-power view of an H&E-stained section of the 9 months of age case (Figure 2(c)) shows acinic cell carcinoma (asterisk), characterized by variable-sized cystic spaces lined by simple or stratified cuboidal epithelium with some papillary projections. Yellow lines separate the tumor (asterisks) and nonneoplastic lacrimal gland (N). The retrobulbar tumor is separated from the eyeball (E) by the skeletal muscle. (b) Higher-magnification view of the boxed area. Variable vacuolated, clear tumor cells mimicking adjacent nonneoplastic serous acinar cells of the lacrimal gland were found. (c) A poorly differentiated, predominantly solid component with a scant glandular pattern is noted in an H&E-stained section of the 17 months of age case (Figure 2(b)). Scale bars: 200 *μ*m.

To characterize the neoplastic eye abnormalities in *Brca1*-mutant mice, we performed histopathological analyses of these tumor tissues. A retrobulbar mass was detected in the seven *Brca1*-mutant experimental animals that exhibited exophthalmoses (Figure 2). The masses showed an expansive growth pattern, and portions of nonneoplastic lacrimal gland were identified at the tumor periphery (Figures 2(a) and 2(c)). All cases were extraocular lacrimal carcinoma, characterized by variable-sized cystic spaces lined by simple or stratified cuboidal epithelium with some papillary projections. A poorly differentiated component composed of intertwining solid or near-solid tubules or stromal hyalinization was noted (Figures 2(b) and 3(c)). Acinar and ductal cells with variable vacuolated and clear features that mimicked adjacent nonneoplastic serous acinar cells of the lacrimal gland and formed solid and microcystic components were also evident (Figure 3). There was no evidence for mitosis, necrosis, pleomorphism, or neural invasion. The final pathologic diagnosis was acinic cell carcinoma arising from the lacrimal gland.

### 3.2. Characterization of Lacrimal Gland Tumors in Brca1-Mutant Mice

**Figure 4:**
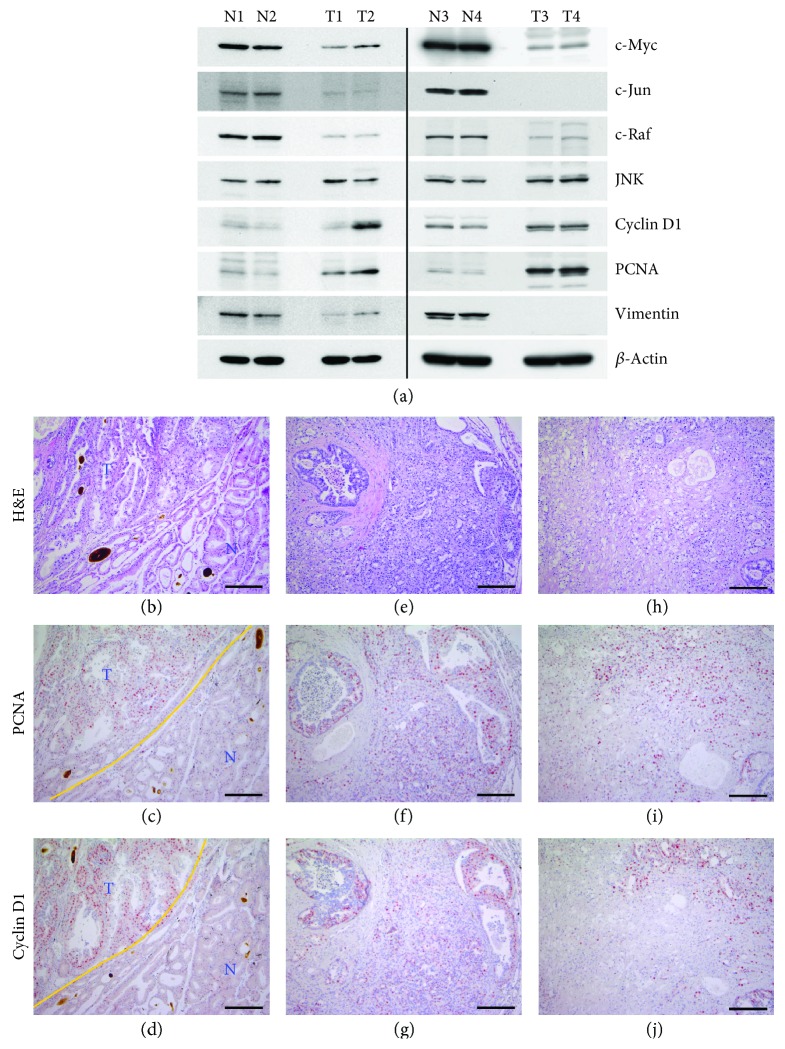
Overexpression of cyclin D1 and increased cell proliferation in lacrimal gland tumors from *Brca1*-mutant mice. (a) Protein expression patterns in lacrimal gland tumors (T1 (10 M), T2 (11 M), T3 (12 M), and T4 (14 M)) from *Brca1^co/co^MMTV-cre* mice compared with the normal tissue (N1, N2, N3, and N4). *β*-Actin was used as a loading control. (b–j) Lacrimal gland sections from tumor areas of *Brca1^co/co^MMTV-cre* mice stained with H&E (b, e, h) and immunostained for PCNA (c, f, i) and cyclin D1 (d, g, j). Yellow lines in (c) and (d) separate tumor (T; upper left) and normal (N; lower right) tissue. Scale bars, 200 *μ*m.

To identify the effector molecules or biomarkers involved in BRCA1-deficient lacrimal gland tumorigenesis, we analyzed various regulatory proteins in *Brca1*-mutant lacrimal tumors and normal lacrimal glands (Figure 4(a)). Expression of the cellular protooncogenes, c-Myc, c-Jun, and c-Raf, was decreased in tumor tissues. Notably, cyclin D1 and PCNA were induced in BRCA1-deficient tumor tissues. To further analyze the relation between PCNA and cyclin D1, we examined the distribution of PCNA- and cyclin D1-positive cells in sections of BRCA1-associated lacrimal gland tumors (Figures 4(b)–4(j)). A comparison of the distribution of these proteins in normal and neoplastic regions showed that PCNA- and cyclin D1-positive cells were abundant among acinar cells in neoplastic areas (Figures 4(c) and 4(d), upper left), whereas such cells were not detected in normal regions (Figures 4(c) and 4(d), lower right). We also identified overlapping PCNA and cyclin D1 positivity in several highly proliferative areas of lacrimal tumor sections (Figures 4(e)–4(j)), suggesting that proliferation of BRCA1-deleted lacrimal gland tumors is associated with accumulation of cyclin D1.

**Figure 5:**
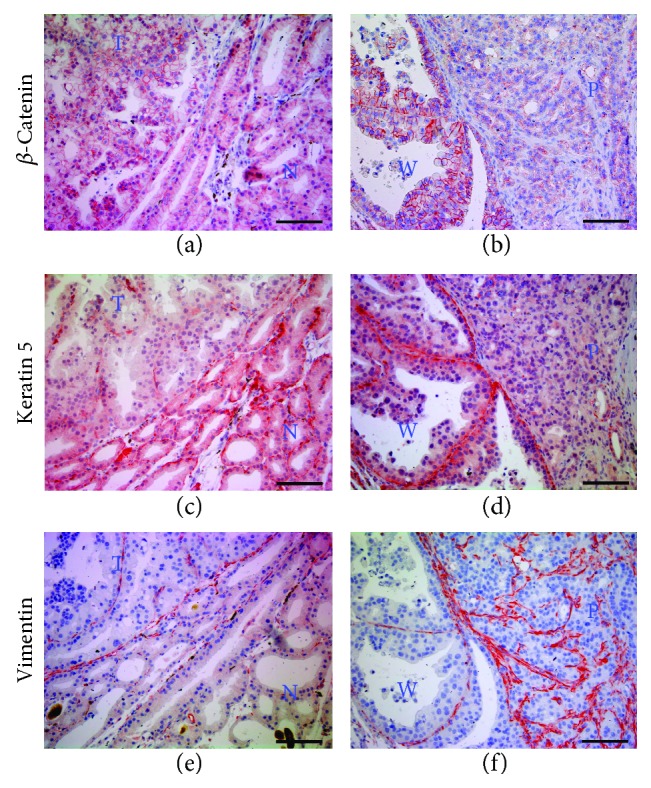
Alteration of skeletal protein distribution in lacrimal gland tumors of *Brca1*-mutant mice. Distribution of *β*-catenin, keratin 5, and vimentin in normal (N; lower right) and tumor-containing (T; upper left) regions (a, c, e) and well-differentiated (W; lower left) and poorly differentiated (P; upper right) regions (b, d, f) of a lacrimal gland. Scale bars: 100 *μ*m.

On the other hand, although specific molecular alterations that characterize acinic cell carcinoma have not yet been elucidated, activations of *β*-catenin and vimentin were reported in previous studies [12–14]. We also examined the distribution of cytoskeletal proteins in tumor tissues of BRCA1-associated lacrimal glands. *β*-Catenin, localized to the plasma membrane of acinar cells, was highly expressed in well-differentiated carcinomas (Figures 5(a), left and 5(b), left) compared with normal tissue (Figure 5(a), right) and poorly differentiated carcinomas (Figure 5(b), right). In addition, basement membrane-localized keratin 5 was highly expressed in the normal region (Figure 5(c), right), and gradually decreased as tumorigenesis progressed (Figures 5(c) and 5(d)). In contrast, distribution of vimentin is abundant in tumor (upper left) than normal region (lower right) (Figure 5(e)) and elevated in poorly differentiated (right) than well-differentiated tumor (left) (Figure 5(f)), suggesting induction of vimentin according to the aggressiveness of the BRCA1-associated lacrimal gland tumor (Figures 5(e) and 5(f)).

Taken together, these findings suggest that BRCA1-associated lacrimal gland tumors share molecular characteristics with BRCA1-deficient breast cancer and that structural proteins exhibit the alteration in level and distributions depend on the progression of cancer.

## 4. Discussion

Germline mutations in the *BRCA1* gene are associated with an elevated risk of breast and ovarian cancer [15]. Approximately 1 in 8 women (~12.4%) will develop invasive breast cancer over the course of their lifetimes, whereas the estimated average cumulative risk for these cancers by age 70 among BRCA1 carriers is 60% [16]. However, the extent of developing other cancers in *BRCA1* mutation carriers is less clear. To investigate the contribution of BRCA1 to breast cancer, mice with *cre*-mediated deletion of *Brca1* in mammary epithelial cells (*Brca1^co/co^MMTV-cre* mice) were developed and displayed the mammary tumor formation after a long latency [10]. Studies performed in the animal facility of the National Cancer Center showed that 15.5 months was required for 50% of *Brca1^co/co^MMTV-cre* mice to exhibit a mammary tumor. Using this same mouse model, we found 7 mice developed a malignant lacrimal gland tumor later in life (9, 10, 11, 12, 14, 16, and 17 months of age).

Surprisingly, although the original goal of developing *MMTV-cre* transgenic mice was to induce targeted deletion of *Brca1* in epithelial cells of mammary glands using cre recombinase, these mice also exhibited tumors in the lacrimal gland. Indeed, previous studies have reported that activation of genes driven by the *MMTV* promoter results in abnormal lacrimal gland phenotypes. For example, mice with an *MMTV-Notch4* transgene not only display mammary gland abnormalities but also exhibit unusual proliferation of immature epithelial cells in the lacrimal gland [17]. *MMTV-vH-ras* mice also frequently show lacrimal gland hyperplasia [18]. These results suggest that the *MMTV* promoter is also actively transcribed in the lacrimal glands.

Acinic cell carcinoma is a rare neoplasm that is nearly always observed in the salivary glands. Risk factors for acinic cell carcinoma are not yet clear but could include previous radiation exposure and familial predisposition [19]. Acinic cell carcinoma in a lacrimal gland tumor was first described by De Rosa et al. in 1986, and the few cases documented since in humans and animals have been shown to exhibit aggressive behavior in association with poor prognosis [20–25]. In the current study, the disease manifested as exophthalmos and was confirmed by radiographic examination and further histopathological and immunohistochemical analysis. Acinic cell carcinoma is a low-grade malignant tumor and further transformed to the high-grade malignancy characterized by the dedifferentiation. The high-grade transformation delineates a more aggressive behavior and is associated with a poorer prognosis than its traditional counterpart. Among the seven cases with lacrimal tumor, three cases were subjected to the histopathological analysis while others were only applied to the Western blotting for a small size of tissue sample. Our histopathological analysis revealed that all the cases of lacrimal abnormalities from *Brca1*-mutant mice were extraocular lacrimal malignant tumor, characterized by variable-sized cystic spaces lined by simple or stratified cuboidal epithelium with some papillary projections. Stromal hyalinization, vacuolated structures, and microcystic components were also identified in acinar cells of the lacrimal gland. Importantly, an exceedingly peculiar variant of this tumor has been described as acinic cell carcinoma with high-grade transformation/dedifferentiation which is characterized by the coexistence of both low-grade acinic cell carcinoma and a high-grade dedifferentiated component, as well as by an accelerated clinical course. In 17 months of age case, a poorly differentiated component composed of intertwining solid or near-solid tubules or stromal hyalinization was noted (Figures 2(b) and 3(c)). In addition, acinar and ductal cells with variable vacuolated and clear features that mimicked adjacent nonneoplastic serous acinar cells of the lacrimal gland and formed solid and microcystic components were also evident in the 9 months of age case (Figure 3). To provide detailed information of the malignancy, we took the representative high-magnification images of each tumor in histopathological analysis and arrange with low-power images (Figure 2). Taken together, data define the tumors arising in the lacrimal glands as acinic cell carcinoma. Our molecular characterization of BRCA1-deficient lacrimal acinic cell carcinoma using immunohistochemistry showed that proliferating acinic cells overlapped with the expression of cyclin D1, revealing a property in common with BRCA1-associated mammary tumors in mice [26]. Our immunohistochemical examination of skeletal proteins also suggested that the appearance and distribution of the immunomarkers, *β*-catenin, keratin 5, and vimentin, are dependent on tumor progression.

In human *BRCA1* mutant carrier, breast and ovarian epithelial cell carcinomas are the predominant abnormalities in whole-body haploinsufficiency. In this study, in the cohort of conditional *Brca1* knockout mice, we identified several acinic cell carcinomas in the lacrimal gland. However, there is no report of lacrimal gland tumor with alterations in *BRCA1* in human. A recent study by Ripamonti et al. described the first case of acinic cell carcinoma of the breast occurring in a carrier of a *BRCA1* mutation [27]. The patient, who had an invasive ductal carcinoma, had a recurrence of a triple-negative acinic cell carcinoma in the contralateral breast 4 years later. Sequencing analyses of both tumors revealed a loss of the wild-type *BRCA1* allele by loss of heterozygosity in both the acinic cell carcinoma and the invasive ductal carcinoma in association with two different somatic TP53 mutations, suggesting that acinic cell carcinoma develops independent from invasive ductal carcinoma. This report also suggests that a germline mutation of *BRCA1* facilitates the development of acinic cell carcinoma as well as ductal carcinoma.

## 5. Conclusion

For the very low incidence of lacrimal gland tumor, few large improvements have been made in its basic research and clinical investigation. In the current study, considering the low incidence of *BRCA1*-associated acinar cell carcinoma and lacrimal gland tumor, *Brca1*-mutant mice were found to be a useful model for investigating tumorigenesis of lacrimal gland tumors and preclinical study for testing treatment efficacy. In addition, our study shows that BRCA1 is involved in homeostasis and genetic stability of the lacrimal gland and provides new insight into genomic instability in organism maintenance and lacrimal gland tumorigenesis.

## Data Availability

The data used to support the findings of this study are available from the corresponding author upon request.
